# “*Once the government employs you, it forgets you*”: Health workers’ and managers’ perspectives on factors influencing working conditions for provision of maternal health care services in a rural district of Tanzania

**DOI:** 10.1186/s12960-015-0076-5

**Published:** 2015-09-14

**Authors:** Dickson Ally Mkoka, Gladys Reuben Mahiti, Angwara Kiwara, Mughwira Mwangu, Isabel Goicolea, Anna-Karin Hurtig

**Affiliations:** Department of Clinical Nursing, School of Nursing, Muhimbili University of Health and Allied Sciences, Dar es Salaam, Tanzania; Department of Development Studies, School of Public Health and Social Sciences, Muhimbili University of Health and Allied Sciences, Dar es Salaam, Tanzania; Department of Public Health and Clinical Medicine, Unit of Epidemiology and Global Health, Umeå University, 901 85 Umeå, Sweden

**Keywords:** Working conditions, Health worker, Governance of human resource for health, Maternal health services, Rural health facilities, Tanzania

## Abstract

**Background:**

In many developing countries, health workforce crisis is one of the predominant challenges affecting the health care systems’ function of providing quality services, including maternal care. The challenge is related to how these countries establish conducive working conditions that attract and retain health workers into the health care sector and enable them to perform effectively and efficiently to improve health services particularly in rural settings. This study explored the perspectives of health workers and managers on factors influencing working conditions for providing maternal health care services in rural Tanzania. The researchers took a broad approach to understand the status of the current working conditions through a governance lens and brought into context the role of government and its decentralized organs in handling health workers in order to improve their performance and retention.

**Methods:**

In-depth interviews were conducted with 22 informants (15 health workers, 5 members of Council Health Management Team and 2 informants from the District Executive Director’s office). An interview guide was used with questions pertaining to informants’ perspective on provision of maternal health care service, working environment, living conditions, handling of staff’s financial claims, avenue for sharing concerns, opportunities for training and career progression. Probing questions on how these issues affect the health workers’ role of providing maternal health care were employed. Document reviews and observations of health facilities were conducted to supplement the data. The interviews were analysed using a qualitative content analysis approach.

**Results:**

Overall, health workers felt abandoned and lost within an unsupportive system they serve. Difficult working and living environments that affect health workers’ role of providing maternal health care services were dominant concerns raised from interviews with both health workers and managers. Existence of a bureaucratic and irresponsible administrative system was reported to result in the delay in responding to the health workers’ claims timely and that there is no transparency and fairness in dealing with health workers’ financial claims. Informants also reported on the non-existence of a formal motivation scheme and a free avenue for voicing and sharing health workers’ concerns. Other challenges reported were lack of a clear strategic plan for staff career advancement and continuous professional development to improve health workers’ knowledge and skills necessary for providing quality maternal health care.

**Conclusion:**

Health workers working in rural areas are facing a number of challenges that affect their working conditions and hence their overall performance. The government and its decentralized organs should be accountable to create conducive working and living environments, respond to health workers’ financial claims fairly and equitably, plan for their career advancement and create a free avenue for voicing and sharing concerns with the management. To achieve this, efforts should be directed towards improving the governance of the human resource management system that will take into account the stewardship role of the government in handling human resource carefully and responsibly.

## Introduction

The human resource for health crisis is one of the concerns for health system performance in many low-income countries. Provision of quality health services such as safe pregnancy and delivery services to mothers is impaired by this crisis, limiting the countries from achieving health and development goals [1].Several studies on human resource for health have described factors responsible for health workers’ shortage in developing countries [2-6]. Underproduction, misdistribution, migration of health workers both within and outside the country and an increased demand for health services with increased population are documented to be the main causes for shortage of health workers. Despite the fact that the causes for the shortage are well known, they are poorly addressed in health-related policies and receive little attention [7-9]. In fact, approaches that address health workers’ shortage mainly focus on production, recruitment and deployment of health workers in remote areas. This overlooks the root causes that affect retention and performance such as personnel motivation, work environment, personnel management system and work organization process [7,8,10-14]. Addressing such root causes for human resource crisis could create good working conditions for efficient service provision.

Understanding the influence of working conditions on health workers’ performance is one of the vital steps towards addressing human resource crisis in developing countries. Researchers and international organizations working on human resource for health have started to advocate for good working conditions as a strategy to achieve better performance among health workers. The World Health Organization (WHO) defines working conditions as the combination of compensation, non-financial incentives and workplace safety [15]. The 2006 World Health Report of WHO emphasizes having a human resource management that motivates, supports and develops health workers to achieve both international and national health goals [15]. In a meeting held in Uganda in 2008, the Global Health Workforce Alliance came up with the so-called Kampala Declaration that pointed out the need for adequate incentives and an enabling and safe working environment for effective retention and equitable distribution of the health workforce [16]. Wyss [9] clearly indicated the impact of poor working conditions as one of the constraints that hinder human resource contribution to the attainment of health-related Millennium Development Goals. Other researchers have commented on the need to establish a positive working environment, keep communication open, provide opportunities for career advancement and recognize and reward hard-working health workers [2,17]. However, for this to work, there is a need for strengthening human resource management systems and consider the role of governance in addressing human resource crisis [18-20]. According to Kaplan and her colleagues, there are eight governance principles that, if applied, could strengthen the management of human resources [21]. These principles include the following: strategic vision, accountability, transparency, information, efficiency, equity/fairness, responsiveness and voice and participation. They further advocate for more researches on how operationalization of these principles can improve health workers’ conditions and their performance in service provision including provision of quality maternal health care services [21].

The need for conducive working conditions on performance of health workers providing maternal health care services cannot be overemphasized. Reduction of maternal deaths through provision of emergency obstetric care, for example, can be achieved only if there are skilled personnel working in an environment where there is adequate drugs and medical supplies to be used when needed [22-25]. Furthermore, inadequacy of equipment and essential drugs combined with high workload and lack of professional retraining are frequently reported factors responsible for low motivation and poor performance of health workers providing services to pregnant women [26,27]. In other studies, health workers perceived that difficult working conditions associated with lack of supervision accounted for their low performance leading to provision of poor maternal care services [28,29]. This shows that, despite the health workers’ crucial role in implementing maternal-health-related policies and, in particular, attainment of the health-related Millennium Development Goals 4 and 5 [30,31], their effectiveness is dependent on the number of factors that influence their working conditions.

### Health workforce in Tanzania

Tanzania, like many developing countries, is facing a severe shortage of health workers despite its high disease burden. The country has a ratio of 1 doctor per 25 000 people and 1 nurse per 10 000 people [32], a figure below optimal ratio recommended by the WHO of 23 nurses or doctors per 10 000 people [33]. Furthermore, assisted birth care by trained health personnel is accessible to not more than half of all pregnant women [34]. The health workers’ distribution is unequal between urban and rural areas, with more serving in urban than in rural or hard-to-reach areas [35]. The need for more health workers in rural areas will increase with the ongoing implementation of the primary health care programme which demands availability of a health facility in each village by the year 2017 [36]. Despite the fact that the primary health facilities serve a large population, especially in rural Tanzania (where more than 80% of the population resides), they are understaffed and mostly run by a low cadre of health workforce with minimal skills [37]. Furthermore, most of such facilities lack the skill mix needed to provide optimal and quality maternal health care.

### Approaches to address the human resource crisis in Tanzania

A number of approaches have been developed to address the challenges of health workers in Tanzania. One such approach is reinvention of the centralized–decentralized approach of recruiting health workers with the intention of improving the distribution of health workers to underserved areas [38]. With this approach, the health workers are recruited by Ministry of Health and Social Welfare (MoHSW) and deployed to respective district councils for employment. At the district level, the deployed staffs are distributed into district hospitals, health centres and dispensaries according to staffing needs. The district councils are responsible for rewarding, promoting, disciplining, training, developing and firing their personnel [39]. While the approach was reported to improve retention of the lower cadre in remote areas, it failed in ensuring retention of highly skilled health workers in these areas [38]. In acknowledging the crisis of human resources in the health sector, the government through the Ministry of Health developed a 5-year strategic plan from 2008 to 2013, stipulating interventions that would address major challenges contributing to shortage. Specifically, this plan focused on, among others, training and development, workforce management, strengthening of leadership and stewardship in executing responsibilities related to the management of human resource for effective services [32].With regard to training, in particular, the government increased enrolment of pre-service students in both medical and nursing schools, aiming at increasing the number of graduate health workers. The government also increased the number of employment permits in recent years resulting in increased number of graduates posted by MoHSW for recruitment by councils from 983 in 2005/2006 to 5720 in 2012/2013 [40].

Despite implementation of this plan and other government efforts, the country still experiences a critical crisis of human resources for health. Meanwhile, existing health workers face constrained working conditions impacting their ability to provide quality maternal health care services as was observed in many health facilities within the country [41,42]. The situation is even more severe in rural Tanzania where the living environment further creates difficulties in both attracting and retaining skilled health workers, leaving many health facilities in these areas understaffed. Additionally, concerns such as those related to delayed promotion, lack of supervision, denied access to training and perceived unfairness in allocation of allowance contributed to low motivation, job dissatisfaction and sometime strikes [17,43,44].

While there is considerable effort from the government in increasing production and deployment of health workers in response to the human resource crisis, a comprehensive understanding of factors that affecting their retention and performance is a vital starting point to effective interventions in the future. Specifically, exploration of how organizational processes and governance arrangements influence the working conditions of staff already in practice is of paramount importance in order to address the human resource crisis comprehensively. Learning from the experiences of the current health workers on their working conditions can better provide empirical evidence to inform future human resource policies and strategic planning that will shape overall management of human resource for health in the country. This study was therefore set to explore the perspective of health workers and managers on factors influencing working conditions for providing maternal health care services in rural Tanzania. The researchers took a broader picture of understanding the status of the current working conditions through a governance lens and brought into context the role of the government and its decentralized organs in handling health workers in order to improve their performance and retention.

## Methods

A qualitative study design was chosen using in-depth interviews with health workers and district health managers, observations and document review to explore factors influencing working conditions for providing maternal health care services in rural Tanzania.

### Study setting

This study was conducted in the Kongwa district in the Dodoma region of Tanzania. The district has typical rural characteristics with a moderate level of socio-economic development and is fairly accessible in terms of transport and communication networks. The major economic activities are agriculture characterized by small farming and livestock keeping. The district has a dry Savannah type of climate which is characterized by low and unpredictable unimodal rainfall, persistent desiccating winds and low humidity. Transport system consists of unpaved roads which are hardly accessible during rainy season. The headquarters which bears the same name of the district is located about 89 km east of Dodoma town. While electricity is available in very few areas, access to clean water is still a problem in many villages. The district has 14 wards, 67 villages and 286 hamlets. The total population of the Kongwa district is 309 973 people as per the 2012 population census projection [45]. The health care system in Kongwa consists mainly of government-owned facilities, with very few health facilities owned by the private sector and non-governmental organizations (NGOs). The government-owned facilities include 1 district hospital located in the district centre, 4 rural health centres and 32 dispensaries. A review of the Comprehensive Council Health Plans and district health reports indicated that by the end of 2012 the district had a total of 327 health workers working in these facilities out of 664 that were required. All these facilities provide antenatal care (ANC), delivery care and postpartum care. Caesarean section is provided in one rural health centre and at the district hospital. As in many other district, women with high-risk pregnancies and those with complications are identified by health workers during ANC at dispensaries and rural health centres and are referred to the district hospital where they can stay at the maternal waiting home (locally known as Chigonela) located at the district hospital while waiting for delivery. The maternal waiting home allows immediate access to emergency delivery at the district hospital, such as caesarean section.

### Data collection techniques

In-depth interviews and observations were conducted during four phases between December, 2011, and May, 2013. In the first phase (December 2011), initial exploratory interviews with five members of the Council Health Management Team (CHMT) resulted in the emergence of issues related to health workers’ shortage that needed further exploration. In the second phase (February-April, 2012), 18 health facilities were visited and structured observations were conducted using an observation checklist focusing on health facility structure (status, source of light, running water, latrines and delivery room), drug and staff house availability and status. The choice of facilities was done to capture the geographical variation of the district and different types of health facilities (1 hospital, 3 health centres and 14 dispensaries) from both central and rural parts of the district. The third phase (February, 2013) involved in-depth interviews with 15 health workers (1 assistant medical officer, 5 nurses, 4 clinical officers, 4 medical attendants and 1 laboratory technician) working in the 18 health facilities that were previous observed by the first author. Selection of informants was purposely done to include different categories of service providers. An interview guide was used with questions to explore informant’s perspective on working conditions focusing on working environment, living conditions, managements’ responses to their claims, career advancement, training opportunities and means they use to communicate their concerns. The guide was developed based on existing literature related to working conditions, prolonged experience of the first author in the study setting and informal discussion with health workers prior to the study. Probing questions on how these issues affect the health workers’ role of providing maternal health care were employed. Follow-up interviews with seven managers directly responsible for human resources management (five members of CHMT, two informants from District Executive Director’s (DED) office) were done during phase 4 (May, 2013) to explore on how health workers’ concerns are dealt at the managerial level.

The first author together with the research assistant collected data, and all interviews were audiotaped. Field notes and memos were written up both during and immediately after the interviews.

Documentary reviews were conducted to supplement the data. Documents reviewed include the Human Resource for Health Strategic Plan (2008-2013), health sector strategic plans, council health planning documents (2008-2012), local-government-related policy documents and Tanzania maternal-health-related policies. These documents were reviewed to gain an insight on the structure and function of the Local Government Authority (LGAs) in relation to human resource for health and how health-related policies and local government’s documents address various health workers issues in rural settings.

### Data analysis

Audiotaped interviews were first transcribed by the first author and translated from Kiswahili to English. The interviews were analysed using qualitative content analysis, following Graneheim and Lundman [46]. The transcripts and field notes were first analysed manually by reading and re-reading to become familiarized with the data. Transcripts from health workers and managers were analysed for identification of text (meaning units) related to informants’ perspectives on provision of maternal care, health workers’ experiences related to working environments, living conditions, dealing with staff welfare and staff management in general. Then codes were extracted from these condensed-meaning units. The codes from health workers’ and managers’ transcripts were further analysed in order to distinguish similarities and differences. Then similar codes were sorted together to form categories reflecting the manifest content of the text.

### Trustworthiness

Trustworthiness in the study is achieved when the findings are worth believing [47]. According to Graneheim and Lundman [46], trustworthiness in a qualitative study is assessed using four criteria namely credibility, transferability, dependability and confirmability. Credibility in this study was ensured through triangulation of different study informants with various experiences who shed light on the research question from a variety of aspects. To enhance credibility and dependability, the data collected from interviews were triangulated with those from field notes, document reviews and observations of health facilities during the analysis process, and categories and themes were shared among the co-authors with different backgrounds and degrees of familiarity with the setting who gave critical comments and suggestions. To confirm that the findings reflected informants’ perspectives rather than researchers’ understanding of the problem, the presented findings were supported by codes and quotes. Transferability was enhanced by describing the study context, process for data collection and analysis.

### Ethical considerations

The study was ethically approved by the Senate Research and Publication Committee of Muhimbili University of Health and Allied Sciences. Permission to conduct the study was given by the Dodoma Regional Administrative Secretary (RAS). Informed consent was obtained after the researchers explained the purpose of the study. Participants were informed of their right to refuse participation in the study and were assured of the confidentiality of the collected information.

## Results

Four categories emerged during analysis of the data that were cross-cut by one broader theme, “Feeling abandoned and lost within an unsupportive system”. This theme refers to a frustrating working environment and the insecure and unsatisfactory living condition that informants perceived as dominant concerns that influence health workers’ working conditions for provision of quality maternal health care services. This, together with their perception of the administration system being irresponsible and bureaucratic and the uncertain future of their career advancement, further left health workers feeling abandoned and lost within the system they serve. Table 1 indicates emerging categories with selected corresponding codes.

**Table 1:** **Selected codes and categories emerged while exploring the perspective of health workers and managers on factors influencing working conditions for providing maternal health care services in rural Tanzania**

**Selected codes**	**Category**
Missing reagents for performing investigations	Frustrating working environment
Delivering using phone torch during night	
Lacking running water at the facility	
Overwhelmed by excessive workload	
Lacking supervision and mentorship	
Not compensated for working overtime	
Committed staff clash with lack of motivation mechanisms	
Lack of decent houses prepared for new employed staff	Insecure and unsatisfactory living conditions
Fear for security during nights	
Anxious over home property during rainy season	
Concern over quality of education for staff children	
Long process in dealing with staff concerns and claims	Bureaucratic and irresponsible system in dealing with health workers’ claims
Lack of transparency in dealing with staff monetary claims	
Irresponsible district administrative system	
Lacking system to air out their concerns	
Lack of forecasting for staff needs	Uncertain vision and plan for staff career advancement and continuous profession development
Unavailable fund for capacity building	
Budget shutdown for training in service staffs	
Unclear staff development plan for low cadre	

### Frustrating working environment

Health workers interviewed expressed frustrations over working environment that handicapped their role of providing quality maternal health care services. From their perspective, the quality of maternal health care services provided is suboptimal. Issues such as missing reagents for investigations in ANC clinics, lack of reliable sources of light during night and lack of running water for cleanliness were viewed by informants to contribute to suboptimal maternal health care services in their facilities. Lack of reagents and equipment for investigations at the facilities caused health workers to fail to conduct comprehensive assessment during ANC visits as recommended for comprehensive focused antenatal care (FAC).The following quote attests to this:

“During ANC, we give health education touching all aspects as described by FAC, but when it comes to investigations, no reagents or equipment to check Hb, [Haemoglobin] or Albumin in urine. Medical store department don’t supply even if we order them. In fact all these are supposed to be done at ANC clinic, but they are not done as recommended in FAC.” (Nurse, Health Centre A).

Some informants from dispensaries explained that mothers often come to deliver during night and reported using a mobile phone torch when conducting delivery because their facilities lack a reliable source of light during the night. This situation was reported to expose mothers, babies and health workers to injuries and infection as observed by one staff:

“See this scar, one time I was conducting delivery during night using mobile phone torch, I couldn’t see the surgical blade I used to cut the umbilical cord and when I was assembling equipment, I cut myself unknowingly, thank God the woman was safe without HIV.” (Nurse, Dispensary C).

Other informants report that their facilities lack clean running water, further compromising cleanliness and increasing chances for contamination. Almost half of the facilities visited for observation did not have clean running water. It was also noted in some facilities that the health workers either buy and store water at the facilities with their own money or ask relatives to come with it from home. Discussion with managers and review of documents indicated that the local government authority, the council, has a mandate to ensure that health services are provided within a well-structured health facility.

Both health workers and managers interviewed expressed their concern over staff shortages which resulted in existing staff being overwhelmed by an excessive workload that makes them exhausted and ineffective in serving women and other patients. This is illustrated in the narrative below from one health worker:

“A lot of work here, sometimes you get confused. If you come in the morning, you work up to 6 pm. I am working in the labor ward, if an emergency comes, I leave the labor ward and go to theatre. Women at the labor ward have to wait until I come out from theatre. Still I have to attend to patients at the reproductive and child health clinic, HIV clinic. It is too much. Sometimes you are at home in the evening eating and you get a call to attend an emergency. You reach a point where you become tired and loose the desire to work anymore. Work becomes a punishment.” (Nurse, Health Centre A).

Dissatisfaction over lack of compensation for extra duties and overtime was expressed by almost all health workers across the different levels of facilities. There was a concern from these informants that lack of overtime allowances would demoralize their commitment to work. Those from dispensaries claimed to be denied overtime allowances despite working day and night conducting deliveries in their facilities as described below:

“They say we don’t have overtime allowances at dispensary level though we are conducting deliveries even during the night hours. I told you, last month 16 babies were born. Out of them, 12 were born during night and in this village many mothers come for delivery from 4 pm and that is overtime. But now, who is going to pay you, how can you claim those allowances and through which system?” (Nurse, Dispensary H).

Moreover, some junior staff reported lacking formal mentorship and technical support when they were employed, denying them the chance to fill the theory–practice gap as described by this respondent:

“When you are employed, you practice according to what you learned at school. But those acting as supervisors are too harsh on us, instead of instructing you, they crash you and create inferiority complex. No mentorship there. You can’t be competent and work confidently in such situation.” (Clinical Officer, Dispensary S)

Some informants from dispensaries were wondering why they are not recognized and acknowledged by the management despite working extensively even during the weekend and holidays. Lack of actual assessment of work being done was reported to be a problem leading to an underestimation of staff workload as described by one respondent:

“There are not any methods used to motivate staff here although we have been fighting for it for so long time. If people consider the number of activities done per one person, they will find that we are doing a lot. The problem is, people don’t do such assessment.” (Nurse, Health centre A).

Despite the introduction of the Open Performance Review Appraisal System (OPRAS), which is a performance appraisal system in the public sector in Tanzania, it was not mentioned during discussions with either administrators or health workers as a means used for assessing workers’ performance. It was also noted from document review of the district planning documents and from discussion with administrators that no clear mechanism is used by the district to motivate existing health workers as a strategy for increasing their retention. Lack of funding from the central government made the district administration fail to pay its health workers their dues in time as one of the district administrators commented:

“We have a delay of funds directed to the district level. Sometimes we lack enough funding from central government. As a result we fail to pay staff their financial claims such as extra duty allowance and subsistence allowance for the newly employed staff.” (Administrator 2).

Other concerns reported by informants were being neglected and lack of care in time of need, especially when a health worker is sick, an act that could lead to diminished staff morale.

The difficult working environment with a shortage of drugs and supplies, unreliable source of light and clean water, lack of mentorship for junior staff and working extra hours uncompensated were reported to demotivate health workers and compromise their role of providing quality maternal care.

### Insecure and unsatisfactory living conditions

Interviewees were dissatisfied by current living conditions, expressing their concerns over lack of decent housing with no social services such as water and electricity. Other informants were concerned about lack of good education for their children as most of the schools found in the village lack teachers risking future academic achievements of their children. One informant commented this way:

“There are no houses here for workers. Not even good houses to rent. The house that I am staying in now, I just live there because there is no alternative. Apart from that, this is not a good environment for my child’s education. No good school here.” (Nurse, Dispensary D).

Furthermore, some informants reported to living far from their working place, endangering their security especially when called to conduct delivery during the night.

“It is four years since this facility started to operate but still there is no housing for the staff. I stay in a house very far from here; I cannot provide service for mothers during night. How will you go such distance? What if you get beaten on the way?” (Nurse, Dispensary H).

The presence of decent housing for staff was perceived by some informants as a symbol of being respected and acknowledged for their valuable service to the community. Lack of houses for staffs was also considered to be one of the reasons some health workers quit and decided not to stay or decided to go and work in other districts where they will find a good living environment.

“Health workers they normally come to our district and go to the village when posted; nurse and, clinical officers, they all go. The issue is, when they arrive there and see the situation, especially the houses, they compare to where they are coming from, and they decide to leave.” (Administrator 2).

Poor living conditions seemed to affect health workers’ concentration to service provision. One informant described how he worries when it rains because of the poor condition of the house he lives:

“Once government employs you, it forgets you especially when it comes to housing. The house I live in now, when it rains and I am at work, I start to think about what is going to happen, because it can wet my mattress; the roof is not good. I shift my concentration from the patient to what might be happening at home.” (Nurse, Dispensary H).

It was noted from the reviewed document that the role of building houses for staff is the responsibility of the LGA and that the councillors (local politicians) should ensure that the staff houses are ready before the staff is brought to the facility. However, the demand for service by the community and influence of local politicians pushes the district health managers to send staff to the newly built health facility without prior preparation of good housing where they can live. One administrator argued the need to prepare and provide good living conditions for staff as a retention strategy.

“One way to improve retention of our health workers in remote areas is to arrange prior for their housing and subsistence allowance. We need to have a good preparatory phase, housing with furniture. In fact, housing for staff should be built at the same time with the health facility and should be within the same compound.” (Administrator 3).

Lack of decent housing, insecurity and unreliable social services such as water and electricity were reported to affect health workers’ social life as well as attraction and retention of health workers to the district.

### Bureaucratic and irresponsible system in dealing with health workers’ claims

Discussions with administrators at the district medical officer’s (DMO) office revealed a number of actors responsible for dealing with staff affairs especially those related to financial claims. While at the facility level, the manager in charge of the facility is the one to approve any staff claims or letters; at the district level, any claims or letters have to go through the DMO’s office before it is sent to the employer, the District Executive Director’s office. Health workers interviewed viewed this process as long and complex, leading to an unnecessary delay in receiving timely feedback to their claims.

Informants also reported on unfairness and lack of transparency in processing payment of their entitled allowances, pointing out that often payment is done to seniors excluding juniors, and those at the remote facilities are unpaid without any explanation. One informant reported this as follows:

“There is unfairness in payment of the money we claim. We claim a lot of money, but we are not given at all. There is discrimination at the district administration, while others get their claims, others like me are not and we don’t understand why”. (Nurse, Hospital).

However, in discussion with district managers, it was noted that the payment of staffs’ financial claims is done using funds received from the central government and that for the past few years they have been receiving very little and that has led to a failure to pay their staff accordingly or to pay them in phases. The quote below from one of the manager attests to this:

“We fail to pay staff’s claims because we don’t have that capacity now. The money that comes from the government is very little. The district’s collected revenue is also not enough. If we get money, we just pay in phases and others have to wait. We normally tell them that the money has not yet come, though not all health workers get that information, in fact information coverage is a challenge here.” (Administrator 2).

Health workers underlined the need of getting feedback for their claims and the need of having a responsible administrative system accountable to health workers’ rights and concerns as it was presented by this informant:

“I am doing my work here, I do my responsibilities, and I deserve my rights for what I have done. Those in administration demand that we work and be responsible, but they forget to meet our concerns and rights. As administrators they are supposed to remember what they have to do for their employees and do it without being reminded or pushed.” (Nurse, Health Centre A).

Moreover, informants reported on the lack of a functioning avenue where they can voice their concerns, leaving them demotivated with persistent and unresolved problems. One respondent described this concern below:

“There is nowhere to speak out our problems. Nowhere! How can you do that? We are afraid and decide to be silent and continue working with our problems. May be we can say this to people like you but otherwise we are avoiding victimization. But for us who are working in the remote areas, they need to find a way to listen to us a bit.” (Nurse, Dispensary H).

It was noted from discussion with managers that there is no formal system currently used for health workers to channel their complaints. Previously, they used to meet with health workers annually, a forum that helped them hear various concerns from health workers and give feedback. It was reported that this is no longer done due to lack of funding to cover the operation costs of such forums. The following quote illustrates this:

“With regards to forums with health workers, they are now not done. We used to have such a meeting annually, but now we don’t have enough funds to cover that.” (Administrator 2).

Generally, informants reported the bureaucratic process in getting the rights they claimed and the irresponsible administration system in handling their affairs. The perceived lack of transparency particularly in paying allowances created a feeling of unfairness among health workers in the district. The lack of formal forum denied health workers an avenue where they can share concerns with their managers at the higher level.

### Uncertain vision and plan for staff career advancement and profession development

Informants view the management as not planning for the needs of new staff and that they establish a facility without ensuring that the necessary enabling environment is in place.

“The government and management here don’t plan ahead of time. They should make sure that the working environment is ok. They have to ensure medical equipment and drugs are there, but most importantly, housing for newly employed staff. When new employees come and find a house, it is hard to leave.” (Nurse, Dispensary H).

Moreover, some informants reported a lack of in-job training that would help raise their capacity in providing up-to-date care. One informant narrated how he failed to manage a certain disease using newly recommended regimes because he was not trained. Most informants interviewed mentioned that they do not attend any training related to emergency obstetric care or lifesaving skills, raising doubts to their competence in providing quality obstetric care in their facilities. Furthermore, some respondents reported uncertainty of their future career progression, leaving them hopeless and discouraged.

“I am staying here and working just because I want to work. Because now, even if you want to go for further studies, the government is not going to pay for you.” (Clinical Officer, Health Centre G).

Discussion with managers revealed that, in previous years, the district was capable to support health workers pursuing further education. However, due to the shortage of fund directed to the district, even the special capacity-building grants, money meant to the support health worker for career development, was now used to cover cost for other important district activities, such as paying sitting allowances during council meetings.

“Support for career development especially for staff who wants to upgrade is a big challenge now. The council has a portion of funding, known as a capacity building grant. However it is used for other important things. Paying councilors during their meetings.” (Administrator, 2).

Managerial informants reported that they have been instructed by the government to remove budget for in-job training from their plan, and they were concerned that this would affect the health workers’ capacity of providing quality maternal service.

“One way to improve maternal health is to train health workers. But now we have been told there should be no budget for training at the district. The government needs to be visionary on how it empowers its health workers.” (Administrator 5).

The need for adequate preparation for new employees both in terms of living and working environment, support for carrier advancement and in-job training were concerns raised by informants and viewed as contributory factors for improving health workers’ performance in providing quality maternal care.

## Discussion

This study has explored factors contributing to working conditions in a rural setting and how these factors affect health workers’ provision of quality maternal health care. We focused on the provision of maternal health care services as health workers’ working conditions are important for ensuring that both maternal deaths and disabilities are prevented. The findings of this study have general implications given the fact that the health workers involved in the study area also provide health services other than maternal health care services. Also, it should be noted that these findings reflect the working conditions in the study area during the study period. However, a number of government initiatives are ongoing both nationally and locally focusing at improving primary health services as stipulated in the Primary Health Services Development Program which ends in 2017 [36].

In this study, it was revealed that the working conditions of rural health workers are compounded by difficult working and living environment, bureaucratic and irresponsible management, unclear career advancement, lack of in-job trainings and formal mechanism for voicing concerns. The compromised working conditions were reported to have direct effects on maternal health care provision, motivation to work and retention of health workers in the study area. While lack of material resources could limit health workers from providing optimal maternal health care; uncertain career advancement, perceived unfairness and lack of transparency in dealing with health workers’ entitled allowances could have significant impact on health workers’ morale and motivation to work and stay as reported in other studies done in Tanzania and elsewhere [41-44,48,49]. Health workers’ motivation is of paramount importance especially in the critical area of maternal health care where providers besides being skilled, needed to be willing and committed to provide quality maternal health care.

Studies done elsewhere indicate a link between constrained working conditions for health workers and poor maternal health care services [26,28,29,49]. Reflecting from these findings, three governance issues in relation to addressing working conditions for the health workforce emerged that need special attention: Firstly, responsibility of the local government in creating safe, attractive and conducive working and living environments; Secondly, the need for a transparent management system to handle health workers’ claims and needs fairly and equitably; Thirdly, the necessity for creation of a free avenue for health workers’ voicing and sharing of their concerns with management.

### Responsibility of the local governments in creating conducive working and living environments for the health workers

Unconducive working and living conditions reported in this study contributed to low morale and under performance of health workers in providing maternal health care. Studies done in Tanzania and elsewhere linked difficult working and living condition with health workers’ low motivation and poor performance [2,10,11,14,17,37,41-43, 48]. Inadequacy of facility infrastructure and unavailability of resources reported in this study played a key role in affecting health workers’ performance. Lack of running water and a reliable source of light in health facilities increased chances of cross infection putting health workers and women using these facilities at risk. This, along with an excessive workload as a result of shortage, unavailability of material resources and lack of supervision further handicapped health workers’ capacity leading to provision of suboptimal maternal care that leave women unsatisfied with the type of care they receive. Mistrust towards health workers and the health system in general become an outcome, and with continued blames received from the community, health worker morale decreased further. Good relationships with service users, recognition and sense of being valued have been considered to contribute in raising health workers’ morale and hence improving their performance [50,51].

Challenges associated with health workers’ working and living conditions as pointed out in this study may be related to the failure of LGAs to fulfil their role of ensuring provision of quality health service in their area of jurisdiction. As a result of decentralization, the local government became responsible in providing and managing primary health services at the level of the dispensary, health centre and district hospital. To fulfil this role, these authorities were obliged to ensure that the health services are provided by adequate and skilled staff working in well-structured health facilities supplied with necessary drugs and other medical supplies. This is important if accessibility to quality maternal health care and reduction of maternal deaths and disabilities are to be achieved. Moreover, the LGAs, through their council, have to ensure that the deployed health workers work in a safe environment and live in decent houses close to social services such as clean water supplies, electricity and good schools for their children. Despite the roles being officially assigned to the local governments, evidence from this study demonstrates the failure of these authorities to fulfil fully their responsibilities of ensuring provision of quality maternal health care services in their areas of jurisdiction. Unclear distinction on service provision between central government and the local authorities, dependency on unreliable funds from the central government and disparity between the responsibilities given and power bestowed to the local government has been documented to contribute to accountability failure of the local governments for their local responsibilities [52-54].

### Transparent management system in handling health workers’ claims and needs fairly and equitably

The perceived lack of a transparent system in dealing with health workers’ monetary claims and unfair reimbursement of allowances were identified as issues that contributed to low morale and poor performance. This could have overall negative effects in provision of quality maternal health care services in this setting, given the fact that effective provision of any health service requires availability of health workers with required skills, positive attitudes and motivation to provide care [55]. Though compromised transparency in accessing training opportunities and promotions did not emerge strongly in our study, other studies done elsewhere report their existence and their impact to health workers’ motivation and performance [17,43]. However, these findings altogether indicate that transparency, equity and fairness are minimally practiced despite being important principles in governing the health workforce [18-21].

The concern for transparency in dealing with health workers’ monetary claims emerged as one of the key issues in this study and was contributed to the lack of feedback to health workers from the management regarding the status of their claims once received at the district level. The payment process for allowances such as on-call allowance, overtime allowance, leave allowance and subsistence allowance given to newly employed health workers were reported to not be transparent and fair. While the health workers submitted their monetary claims formally with relevant documents for evidence, no written feedback is given in return by the management, leaving health workers uncertain about the fate of their claims. This prompted health workers to make frequent physical follow-ups of their claims at the district, leaving health facilities and patients unattended. Given the shortage of health workers that already exists, this further compromised the availability of maternal health care provided in these facilities as a result of providers’ absenteeism. The lack of a well-established information system and effective communication channels between management and workers has been associated with work dissatisfaction resulting to frequent work strikes among health workers [17].

Close to this is the issue of fairness with regard to reimbursement of call allowances and other forms of financial claims. Participants in this study perceived the existence of partiality in payment of allowances, claiming that once money for reimbursement arrived, it is given only to workers working at the district hospital and seniors, leaving others at lower facilities and juniors without any clear explanation. A lack of equity and fairness in issues related health workers’ allocation of allowances, access to training and promotion and recognition has been documented in other studies and linked to contributing factors for attrition particularly in middle-level workers working in remote areas [19-21,43].

### Creation of a free avenue for health workers’ voicing and sharing their concerns

A free avenue and formal system that allows health workers to voice and share their concerns with the management was lacking and is one of a governance issue that emerged in this study. This study found that, despite having a number of problems that affect their working conditions, there was no avenue where health workers could share freely their concerns with the management. Furthermore, the fear of being victimized by the management was found to limit some health workers to voice their concerns with regard to either working environments or health workers’ welfare. As a means to avoid victimization, the decision to remain silent and continue working was adopted by some health workers. This could further worsen working conditions as managers would miss useful information to improve the situation. Voicing, which basically allows the employee to bring to managers’ attention a problem on the ground, has a key contribution in improving organization process, work system and employee morale [56]. Meanwhile, health workers who are also acting as managers of dispensaries and health centres were concerned that even the health plans that they normally develop and send to the district are not incorporated in the district comprehensive health plan which theoretically should reflect health needs evolved from all facilities found in the district. The lack of a clear forum for health workers to share their concerns with management resulted in persistence of unresolved problems which hampers health workers’ productivity and affects their general performance. Unresolved problems and a poor management communication system have been associated with work dissatisfaction and lower workers’ motivation [17]. The findings from this study suggest the need for creating a culture within the human resource management system where health workers could voice their concerns appropriately. This would be an important step towards improving good governance of human resources and therefore better performance. Voicing and participation is now considered to be fundamental principles of good governance [21] although its application in human resource management is not yet clear. However, the creation of voicing mechanisms needs to be done within an overall strengthening of the wider human resource management systems and structure at relevant levels.

### Study limitation

This study revealed governance-related factors affecting health workers’ role of providing quality maternal health care in rural health facilities. However, exploring informants’ possible solutions and the way forward for improvements would better inform human resource managers and policy makers. Furthermore, the study was done in one district and involved a small number of participants, and hence, these findings have limited generalizability. However, many rural districts share similar settings and that the human resource for health management system at the district level is the same throughout the country. Therefore, the insights gained from this study are transferable to other similar settings in Tanzania.

## Conclusion

Health workers in rural settings are crucial in providing maternal health care services and primary health care to the rural population in Tanzania. Generally, provision of quality maternal health care depends on their presence and availability of well-equipped health facilities. However, difficult working conditions handicap health workers’ capacity, lower their morale and thereby affecting their overall performance of providing quality maternal health care. The government and its decentralized organs should be accountable to create conducive working and living environments. It should also respond to health workers’ claims fairly and equitably, plan for their career advancement and create free avenues for voicing and participation in the decision-making process. To achieve this, efforts should be directed towards improving governance of the human resource management system that will take into account the stewardship role of the government in handling human resources carefully and responsibly to ensure the wellbeing of health workers. Governance-related issues that emerged in this study should be taken into consideration when developing and implementing policies of human resources for health. First, the central government’s organs which are responsible for human resource should work closely with LGAs to ensure that health workers are well handled, motivated and supported. Second, an effort should be made at local government levels to establish and operationalize a transparent human resource management system that will facilitate fairness and equity in dealing with health workers’ financial claims, promotion and career development opportunities. Third, a good communication system should be established throughout the human resource management system to enable health workers to share their concerns freely with the management as this will help minimize tension among health workers from chronic irresolvable concerns.
